# Dr. R. B. Winder

**Published:** 1894-08

**Authors:** 


### Obituary.

Died, July 26th at his home in Baltimore, Md., after a protracted nervous affection, a result of the grip, Dr. R. B. Winder.

Dr. Winder has been for many years connected with the Baltimore Dental College and has been for most of the time its dean. He was a man of energy and more than ordinary ability, and had a commanding influence in the dental profession.

The loss of such a man makes a void that is not easily filled.

